# Transcriptomic Study on Ovine Immune Responses to *Fasciola hepatica* Infection

**DOI:** 10.1371/journal.pntd.0005015

**Published:** 2016-09-23

**Authors:** Yan Fu, Andreas L. Chryssafidis, John A. Browne, Jack O'Sullivan, Paul A. McGettigan, Grace Mulcahy

**Affiliations:** 1 UCD School of Veterinary Medicine, University College Dublin, Belfield, Dublin, Ireland; 2 UCD School of Agriculture and Food Science, University College Dublin, Belfield, Dublin, Ireland; 3 UCD Conway Institute of Biomolecular and Biomedical Research, University College Dublin, Belfield, Dublin, Ireland; University of Zurich, SWITZERLAND

## Abstract

**Background:**

*Fasciola hepatica* is not only responsible for major economic losses in livestock farming, but is also a major food-borne zoonotic agent, with 180 million people being at risk of infection worldwide. This parasite is sophisticated in manipulating the hosts’ immune system to benefit its own survival. A better understanding of the mechanisms underpinning this immunomodulation is crucial for the development of control strategies such as vaccines.

**Methodology/principal findings:**

This *in vivo* study investigated the global gene expression changes of ovine peripheral blood mononuclear cells (PBMC) response to both acute & chronic infection of *F*. *hepatica*, and revealed 6490 and 2364 differential expressed genes (DEGS), respectively. Several transcriptional regulators were predicted to be significantly inhibited (e.g. *IL12* and *IL18*) or activated (e.g. *miR155-5p*) in PBMC during infection. Ingenuity Pathway Analysis highlighted a series of immune-associated pathways involved in the response to infection, including ‘Transforming Growth Factor Beta (TGFβ) signaling’, ‘Production of Nitric Oxide in Macrophages’, ‘Toll-like Receptor (TLRs) Signaling’, ‘Death Receptor Signaling’ and ‘*IL17* Signaling’. We hypothesize that activation of pathways relevant to fibrosis in ovine chronic infection, may differ from those seen in cattle. Potential mechanisms behind immunomodulation in *F*. *hepatica* infection are a discussed.

**Significance:**

In conclusion, the present study performed global transcriptomic analysis of ovine PBMC, the primary innate/adaptive immune cells, in response to infection with *F*. *hepatica*, using deep-sequencing (RNAseq). This dataset provides novel information pertinent to understanding of the pathological processes in fasciolosis, as well as a base from which to further refine development of vaccines.

## Author Summary

*Fasciola hepatica* (liver fluke) is not only of major health, welfare and economic importance in ruminants, but also an emerging zoonosis. This parasite is sophisticated in manipulating the host’s immune system to benefit its own survival. In this study we investigated global gene expression changes of the primary innate/adaptive immunity-related cells (peripheral blood mononuclear cells) from sheep pre- and post- infected with *F*. *hepatica*, which revealed the underpinning mechanisms behind various aspects of fluke-induced immunomodulation, including fibrosis, nitric oxide production, regulation of Toll-like receptors, apoptosis of immune cells, and Th17 differentiation. We hypothesis that activation of pathways relevant to fibrosis in ovine chronic infection, may differ from those seen in cattle. The dataset provided here provides information pertinent to understanding of the immune response to sheep fasciolosis. Due to the lack of studies on human fasciolosis, this information is also valuable for exploring human immune response to *F*. *hepatica* infection, as well as for further refine development of vaccines.

## Introduction

The trematode parasite *Fasciola hepatica* (liver fluke) is the causative agent of a global disease (fasciolosis) that is of major health, welfare and economic importance in domestic animals (cattle, sheep) [1]. This parasite can also infect and complete its lifecycle in a wide range of other mammals including humans [2]. Fasciolosis is currently recognized as a major food-borne zoonosis, since human infection cases are distributed broadly, including South America, the Middle East, and Asia [3–5], where it is estimated that 2.4 million people are infected every year, and 180 million people are at risk of infection [2, 6, 7].

Fasciolosis is initiated when the definitive hosts ingest vegetation or water contaminated with an encysted larval parasite (metacercariae). Following hatching from the cyst in the small intestine, the newly excysted juveniles (NEJ) penetrate through the intestinal wall and into the liver. Usually from 4–6 days post infection (acute stage of fasciolosis), the immature flukes migrate through the hepatic parenchyma, and their burrowing and feeding behaviors results in traumatic tissue damage (hemorrhage) and later repair through fibrosis during the next 5–6 weeks. The chronic stage is typically established since the flukes reach the biliary ducts where they mature to adults by 8–10 weeks post infection [8].*F*. *hepatica* has a wide definitive host range, widespread geographical distribution and ability to survive long term in the host, which depends on sophisticated methods of modulating host immune responses to benefit parasite survival [9]. Some currently known methods employed by the parasite include polarization towards a Th2 response, suppression of Th1/Th17 responses, alternative activation of macrophages (characterized by high arginase activity and low iNOS levels), induction of eosinophil apoptosis, and inhibition of dendritic cell maturation (summarized in [9–11]). However the cellular and molecular mechanisms involved in these phenomena are not fully understood. Some efforts have been made to explore gene expression changes in host liver followed by *F*. *hepatica* infection, and these studies yielded important information on the molecular mechanisms involved in host physiological and pathological changes [12, 13]. The main transcriptomic changes identified by these studies were associated with host metabolism, tissue-repair, liver injury and hepatic toxicity; however gene expression changes related to immune response were very limited.

In terms of control strategies against *F*. *hepatica*, triclabendazole (TCBZ) is currently the first choice as a fasciolicide [14]. However, the development of resistance to this compound across Europe, Australia, and in some countries of South America [15–18] has raised concerns about the sustainable control of fasciolosis into the future. Therefore, a ‘greener’ alternative option for control, such as vaccination, is urgently needed. In the last decades, great efforts have been made to identify and test several *F*. *hepatica* antigens as vaccine candidates. For instance, cathepsin L1 proteinase plays multiple key roles in the physiological activity and parasitism of *F*. *hepatica* including digestion of blood, penetration of host tissue, evasion of host humoral immunity, egg production and immunosuppression [19, 20]. A recombinant mutant version of this protein, rmFhCL1, has shown protection against *F*. *hepatica infection* in cattle [21]. However, the current vaccine candidates, although showing promise, have not always been consistent in the degree of protection elicited. Monitoring gene expression changes of host immune cells during a vaccine trial would be useful to understand the vaccine effect. In addition, a more complete understanding of the ways in which *F*. *hepatica* infection modulates the immune response will also benefit the rational design of vaccines which may be able to overcome these immunoregulatory effects.

Peripheral blood mononuclear cells (PBMC) consist of several cell types such as T cells, B cells, Natural Killer Cells and monocytes, which play a crucial role in both innate and adaptive immune response to *F*. *hepatica* infection. We hypothesized that analysis of the transcriptomic changes in the PBMC in response to vaccination and infection would reveal the molecular mechanisms underlying the immune response induced by the vaccine and host-fluke interaction. In this study, we conducted a vaccine trail on sheep and monitored the transcriptomic changes of PBMC induced by vaccine and *F*. *hepatica* infection. Although the immunization did not induce any detectable changes at transcriptional level, we identified a large number of DEGs induced by acute and chronic stage of *F*. *hepatica*, respectively. The present study is one of the first global transcriptomic analysis of ovine PBMC, in response to vaccine and infection with *F*. *hepatica* using RNA sequencing (RNAseq), and provides important new information that enhances our understanding of fasciolosis from an immunological perspective.

## Methods

### Experimental animals and trial design

**Fig 1:**
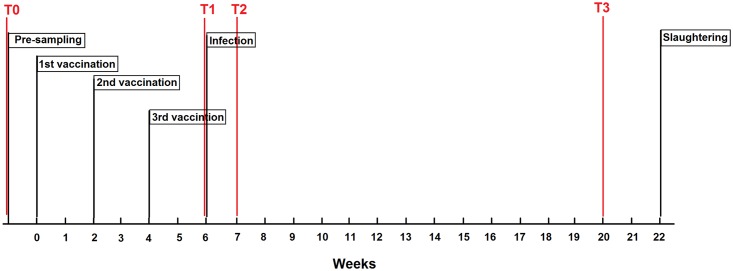
Timeline of the experimental vaccination trial. Experimental procedures were carried out under license from the Health Products Regulatory Authority (Project Authorization Number: AE18982/P010) and after ethical review by the University College Dublin (UCD) Animal Ethics Committee.

Six-month-old lambs (n = 8) used in this study were obtained from the flock at UCD Lyons Research farm, and verified by serology and faecal egg counts as free from *F*. *hepatica* infection. Animals were housed under normal husbandry conditions in fluke-free sheep-pen at UCD Lyons Research Farm. Briefly, three doses of experimental *F*. *hepatica* vaccine, a mixture of 200 μg rmFhCL1, a recombinant protein *F*. *hepatica* Cathepsin L1 (provided as a gift from Professor John Dalton, McGill University), and the adjuvant Montanide^™^ ISA 70 VG (gently provided by SEPPIC), was administered to four lambs (Animal IDs are V1, V2, V3, and V4)at two-weeks intervals (Fig 1). One week after the last vaccination, these animals plus four controls (Animal IDs are C1, C2, C3, and C4)were orally infected with 90 *F*. *hepatica* metacercariae each (Baldwin Aquatics, Inc). The effect of the vaccine was evaluated based on the measurement of fecal egg count (FEC) at 10 and 16 weeks post infection (wpi), and fluke burden (FB) at 16 wpi.

### Ovine PBMC isolation and RNA preparation

Blood was collected into heparinized tubes for each animal at four time-points: pre-vaccination (Time-point 1, T0), pre-infection (T1, just right before inoculation of *F*. *hepatica* metacercariae), 1 week post infection (T2), and 14 weeks post infection (T3). PBMC were isolated from heparinized blood immediately using Histopaque (Sigma). Briefly, 9 ml heparinized blood was diluted in 9 ml of complete medium (RPMI GLUTAMAX, 1% non-essential amino acids, 10% foetal calf serum (FCS), 1% Penicillin Streptomycin). The diluted blood was added to a Leucosep tube with 15 ml Histopaque (Sigma). After centrifuging at 1304 x g for 10 min without the brake, the white PBMC layer was collected from the filter and washed in medium without FCS. The cell pellet was then re-suspended in 1 ml red cell lysis buffer for 1 min. Following two washing steps, the pellet was finally re-suspended in 4 ml complete medium. Cells were counted and their viability checked by trypan blue staining.

Total RNA was extracted from PBMC immediately by using E.Z.N.A Total RNA Kit (Omega), according to the manufacturer’s’ instructions. Purified RNA was treated with RNA-free DNase I (Qiagen). RNA quantity was evaluated using Nanodrop 1000 spectrophotometer (Thermo Fisher Scientific), and RNA quality using an Agilent 2100 Bioanalyzer with an RNA 6000 Nano LabChip Kit (Agilent Technologies). Only samples with A260/280 ratios > 2.0 and RNA Integrity Numbers (RIN) of ≥ 7.8 were used for sequencing.

### RNAseq library preparation and sequencing

Thirty-two RNAseq libraries were prepared using TruSeq Stranded mRNA Sample Preparation Kit (Illumina) following the manufacturer’s instructions. The concentration of each amplified library was measured using Qubit assays (Life Technologies) and the size distribution was assessed on a Bioanalyzer using the DNA1000 kit (Agilent Technologies). Library concentration was normalized to 10nM and pooled for multiplex sequencing. The thirty two RNA-seq libraries were prepared in 4 pools and delivered to the Research Technology Support Facility, Michigan State University, for sequencing. After quantitation and validation by Qubit (Life Technologies) and quantitative real-time PCR (KAPA Biosystems) each pool was loaded on a single lane of an Illumina HiSeq 2500 Rapid Run flow cell (v1). Sequencing was performed using TruSeq Rapid SBS reagents (Illumina) in a 2x100bp (PE100) format.

All RNAseq data has been made available via the NCBI GEO repository [22], under the accession number GSE71431.

### RNAseq analysis

The first stage in sample processing was quality evaluation of the unprocessed FASTQ files using the software FastQC v0.10.0. After filtering out adapter sequence reads and removing poor quality reads, the clean fastq files were aligned to the sheep genome (Oar v3.1, available in the ENSEMBL website [23]) using the alignment software STAR [24] version 2.3.0e_r291 (the parameter used is described in S1 Table). The gene annotation for the sheep genome (Oar3.1 Ensembl release 79) was downloaded from ENSEMBL. After alignment, the gene counts were generated using the software featureCounts [25] from the Subreads software, package version 1.3.5-p4. Transcripts per million (TPM) were calculated based on a simple library size normalization.

### Bioinformatics analysis

In order to visualize the overall structure of the data and to identify potential outliers or mislabeled samples of the data, Principal Components Analysis (PCA) and Between Groups Analysis (BGA) was carried out. The BGA plots were generated using the MADE4 package [26] in an R/Bioconductor [27]. Differential gene expression was calculated using the Limma Voom package [28]. Normalization factors were calculated from the raw counts using EdgeR [29]. The design matrix was generated using Puma [30] fitting main effects of “group” (vaccinated or control) and time along with interactions of “group” with “time”. The “animal ID” was treated as a random effect and “time” was treated as a discrete variable. The empirical Bayes function in Limma [31] was used to determine the significantly expressed genes. A false discovery rate (FDR) of 5% was chosen as a cut-off. The lists of differentially regulated genes for different comparisons were annotated using the R/bioconductor BiomaRt package [32]. Functional pathway analysis was conducted through the use of IPA (Ingenuity Pathway Analysis, QIAGEN). Since the sheep is not currently a supported species in IPA; human orthologues for the relevant sheep genes were used. The human orthologue information was downloaded from BiomaRt and merged with the significant genes. In cases where a single sheep gene was annotated to multiple human genes just one human orthologue was retained for the purposes of pathway analysis. The lists of DEG were used as the input for IPA analysis. ESEMBL ovine ID numbers for genes mentioned in the text were list in Table 1. The curated canonical pathways from Ingenuity Knowledge Base that were enriched in the DEG dataset were determined by using a right-tailed Fisher’s exact test. The enrichment p-value is calculated by assessing the probability of a pathway being randomly selected from all of the curated pathways, and a cut-off of p-value ≥ 0.05 was used as the threshold for significant pathways. The IPA “upstream analysis” feature was used to analyze the upstream regulators. The Ingenuity Knowledge Base was used to predict the expected causal effects between upstream regulators and DEG targets. By calculating an overlap p-value and an activation z-score, the analysis gives a prediction of the status of the upstream regulator.

**Table 1:** List of ID numbers for genes mentioned in the text.

Gene Name	ESEMBL Ovine Gene ID
A2M	ENSOARG00000000950
ACTB	ENSOARG00000003021
ATP5G1/ATP synthase	ENSOARG00000006653
B2M	ENSOARG00000003782
CASP3	ENSOARG00000007472
CCL2	ENSOARG00000009627
CCR2	ENSOARG00000014236
CD28	ENSOARG00000018277
CD40	ENSOARG00000008369
COL1A1	ENSOARG00000004871
CYCS	ENSOARG00000025169
MAPK1/ERK1/2	ENSOARG00000015529
FAS	ENSOARG00000014866
GAPDH	ENSOARG00000007894
CSF2/GM-CSF	ENSOARG00000015430
GUSB	ENSOARG00000001578
HSPB1/HSP27	ENSOARG00000013748
IFNG	ENSOARG00000001958
IFNGR1	ENSOARG00000000510
IL10	ENSOARG00000006292
IL10RA	ENSOARG00000007625
IL15	ENSOARG00000012119
IL17F	ENSOARG00000014066
IL17RC	ENSOARG00000005521
IL18	ENSOARG00000016193
IL23A	ENSOARG00000009246
IL23R	ENSOARG00000011083
IL27	ENSOARG00000003180
IL4	ENSOARG00000014896
IL6	ENSOARG00000012021
NOS2/iNOS	ENSOARG00000016744
JAK2	ENSOARG00000013480
MAP2K4/MKK4	ENSOARG00000015404
novel gene (CXCL5)	ENSOARG00000014592
novel gene (TNFRSF10A/B)	ENSOARG00000017294
SERPINE3/PAI-1	ENSOARG00000008852
PGK1	ENSOARG00000018803
PPARA/PPAR-α	ENSOARG00000019427
RORC	ENSOARG00000021060
RPL19	ENSOARG00000010582
SKI	ENSOARG00000000230
SMAD3	ENSOARG00000018276
SMAD4	ENSOARG00000004662
SMAD7	ENSOARG00000003809
STAT1	ENSOARG00000013903
TBP	ENSOARG00000005142
TGFΒ1	ENSOARG00000007468
TLR1	ENSOARG00000000538
TLR10	ENSOARG00000000520
TLR4	ENSOARG00000005792
TLR5	ENSOARG00000005017
TLR6	ENSOARG00000000552
TLR7	ENSOARG00000011288
TNF	ENSOARG00000008333
TNFRSF1A	ENSOARG00000025177
TNFRSF1B	ENSOARG00000017205
TNFRSF21	ENSOARG00000011638
TNFRSF25	ENSOARG00000012170
TNFSF12	ENSOARG00000000747

### Quantitative real-time PCR validation

Fifteen differentially expressed genes (DEGS) involved in immunological or signaling pathways were selected for validation using quantitative real-time PCR (qPCR). cDNA was synthesized from 250ng total RNA from each sample used for RNAseq library preparation using the High-Capacity cDNA Reverse Transcription Kit (Applied Biosystems), according to the manufacturer’s instructions. In a 20.0 μl reaction, the cDNA was diluted 1:20 with distilled water prior to use. Primers were designed based on the corresponding cDNA sequences (obtained from ESEMBL), and primer efficiencies were determined using a 1:4 dilution series over 7 points, and efficiencies for all primers were between 95–110% (S2 Table). RT-qPCR was performed using SYBR Green Master Mix (Applied Biosystems) on a 7300 Real-Time PCR System (Applied Biosystems). 20μl reaction volume contained 5μl diluted cDNA samples (or appropriate controls), 10μl of SYBR Master Mix and 300 nM final concentration of each primer. RT-qPCR reaction was performed as following cycling parameters: 10 min at 95°C (heat-activation step); 40 cycles of 15 sec at 95°C, 1 min at 60°C. The specificity was confirmed by melt curve analysis. Using the GeNorm algorithm within the qBase+ software package (Biogazelle) the stability of 8 potential reference genes (including GUSB, ATP, PGK1, GAPDH, B2M, ACTB, TBP and RPL19) was assessed (see the corresponding gene IDs in Table 1). RPL19 and TBP were shown to be the most stability expressed (M<0.15) [33]. Calibrated normalized relative quantities (CNQR) were calculated using the qBase+ software [34]. Normal distribution of fold-change values was checked using the Shapiro-Wilk test in the SPSS statistical package (IBM Corp). Two-tailed paired sample t-tests was used to assess statistically significant gene expression fold-changes.

## Results

### No protection was observed in vaccinated animals

There was no significant difference in faecal egg count (FEC) or fluke burden (FB) between vaccinated and control animals, indicating no protective effect elicited by vaccination in this trial (S1 Fig).

### Summary statistics of RNAseq data

The 32 RNAseq libraries (representing vaccinated (n = 4) and control (n = 4) groups from 8 animals at four time-points T0-T3) were sequenced on an Illumina HiSeq 2500 platform and generated mean values per library of 20.3 million paired-end reads, of which 16.0 million reads (81.8%) uniquely mapped to ovine genome (S3 Table). Post alignment quality control (QC) analysis shows no evidence of poor quality or outlier libraries (S2 Fig), and aggressive normalization (e.g. quantile normalization) was not required.

In total 21236 genes are annotated in the Ensembl sheep gene annotation. Genes having a raw count of fewer than 10 reads in fewer than 4 samples were filtered out and removed from the analysis. A total of 13309 genes were retained and used for subsequent differential expression analysis.

### The PBMC transcriptome shifts in response to infection

**Fig 2:**
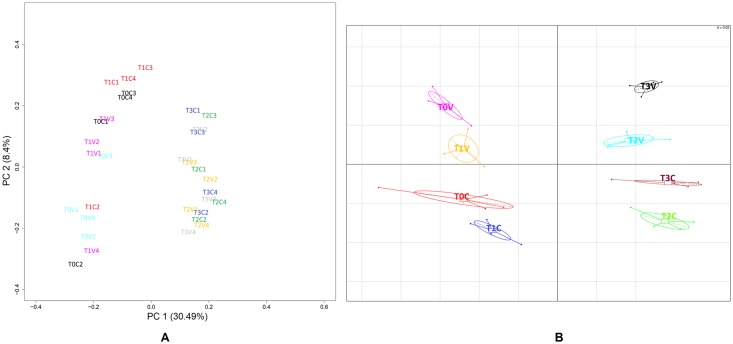
PCA and BGA analysis. (A) Principal component analysis (PCA). The 32 samples are projected onto the 2D plane such that they spread out in the two directions that explain most of the differences. The x-axis, so called the first principal component (PC1), is the direction that separates the samples the most. The y-axis, so called the second principal component (PC2), is an unrelated direction (it must be orthogonal to the first direction) that separates the data the second most. The percentage value in the axis label refers to the percent of the total variance that is contained in the direction. Each sample is represented as time points (T0-T3) followed by its animal ID. For example, T3V3 represents the transcriptome sample from vaccinated lamb V3 at time point 3 (14 wpi). (B) The between groups analysis (BGA) plot based on overall gene expression profiles of different groups in response to *F*. *hepatica* infection over the period of time. V and C represent vaccinated and control group respectively and their time point (0–3) is separated by a dot in each group.

Both PCA and BGA were performed in order to carry out a preliminary separation of samples. The main source of variation between the samples is infection as can be seen from the PCA plot in (Fig 2A). The first principal component separates pre-infection samples (T0 + T1) from post-infection samples (T2 + T3). There is a trend on the second axis with the vaccinated animals shifted slightly along the axis compared to the controls. There is however a lot of overlap between the groups. Using the supervised clustering of BGA (between groups analysis) the same general trends are evident i.e. the biggest effect is the infection effect with T0+T1 and T2+T3 clustering together (Fig 2B). Further differential expression analysis identified very few DEGS between vaccinated and control groups for each time-point individually. Only 12 DEGS were detected at T2, and none was found at any other time-points. When the three post-vaccination time-points (T1-T3) were combined together for this analysis, 36 DEGS (S4 Table) were identified between vaccinated and control animals, due to the increased power given the bigger sample sizes. However manual inspection of these ‘vaccination effect’ genes showed that these 36 genes were systematically different in these animals and were also differentially expressed in the time-point 0 (T0) samples. The samples designated vaccinated (V) were from animals infected with *F*. *hepatica* metacercariae at time-point 1 (T1) therefore there is no experimental difference between control and vaccinated animals at T0. It is likely that these differences instead represent inter-animal variation (likely to be expression quantitative trait loci, eQTLs) and are not relevant to the vaccination effect. Therefore the 36 DEGS representing inter-animal variation were excluded from further analysis.

### Differentially expressed genes and qPCR verification

Since the biggest effect was infection of *F*. *hepatica* rather than vaccination, and in order to increase the statistical power and accuracy for the subsequent analysis [35], we combined the vaccinated and control samples together from each time point, giving a sample size of 8 biological replicates per time-point. To explore the gene expression changes in the acute stage of infection, we compared the data from T2 with T1, and obtained 3134 and 3356 significantly up-/down- regulated DEGS respectively (FDR-adjusted *p*-value ≤ 0.05) (S5 Table). In addition, 1248 significantly up-regulated and 1116 significantly down-regulated DEGS were detected in T3 compared to T2 (S5 Table), which represent the transcriptomic diversity between chronic and acute stage of infection. Notably, the DEGS from both comparisons were all equally distributed between up- and down-regulated. This differs from other transcriptomic studies based on liver tissue from animals infected with *F*. *hepatica* in which the DEGS were biased to up-regulation [12, 13].

**Fig 3:**
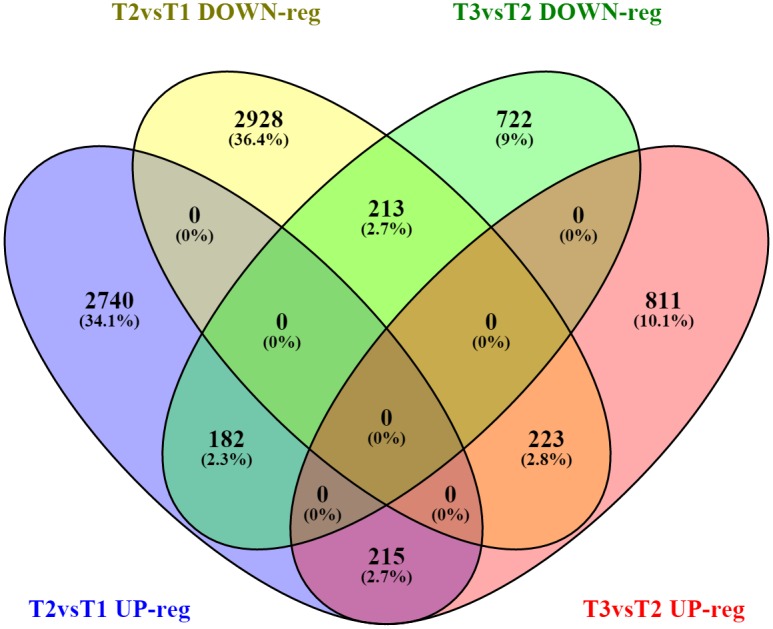
Venn diagram showing the numbers of DEGS identified from T2vsT1 and T3vsT2. Overlap comparison of DEGS from two comparisons (T2vsT1 and T3vsT2) according to direction of expression. Sets of up- / down- regulated genes of T2vsT1 are represented in blue and yellow, up- / down- regulated genes of T3vsT2 in pink and green.

DEGS from T2vsT1 and T3vsT2 were compared according to the direction of expression (Fig 3). 5398 DEGS (2740 up-regulated and 2928 down-regulated) were observed in T2vsT1 only. 1533 DEGS (811 up-regulated and 722 down-regulated) were detected uniquely in the comparison of T3vsT2, demonstrating that gene expression level was significantly regulated by chronic rather than acute *F*. *hepatica* infection. 428 DEGS (215 up-regulated and 213 down-regulated) were observed as occurring in both T2vsT1 and T3vsT2, and displayed the same direction of expression. 182 genes were up-regulated at the acute stage but subsequently down-regulated at the chronic stage of infection, while 223 genes were oppositely regulated over the time course.

S2 Table summarizes the results of qRT-PCR data for the 15 selected DEGS from several canonical pathways of interest. Although the fold change values for the expression of some genes measured by RNAseq or qRT-PCR were quite different, the gene expression patterns of all DEGS in terms of fold-change direction and statistical significance were reproducible by qPCR analysis.

### Functional pathway analysis on DEGS

**Fig 4:**
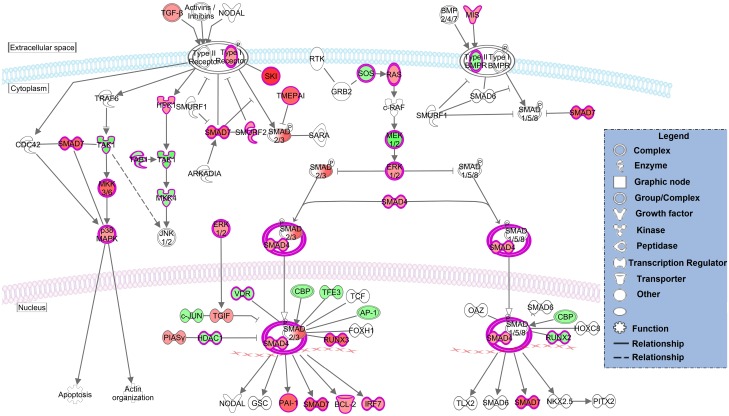
TGF-β signaling pathway. The *TGFB* signaling pathway is represented with gene expression (log2 fold-change) values overlaid. Red shading indicates increased expression in PBMCs at T2 compared to T1. Green shading indicates decreased expression in PBMC at T2 compared to T1. Color intensity indicates expression level. White and grey shading indicates no significantly differential expression, and filtered out due to low expression respectively.

**Fig 5:**
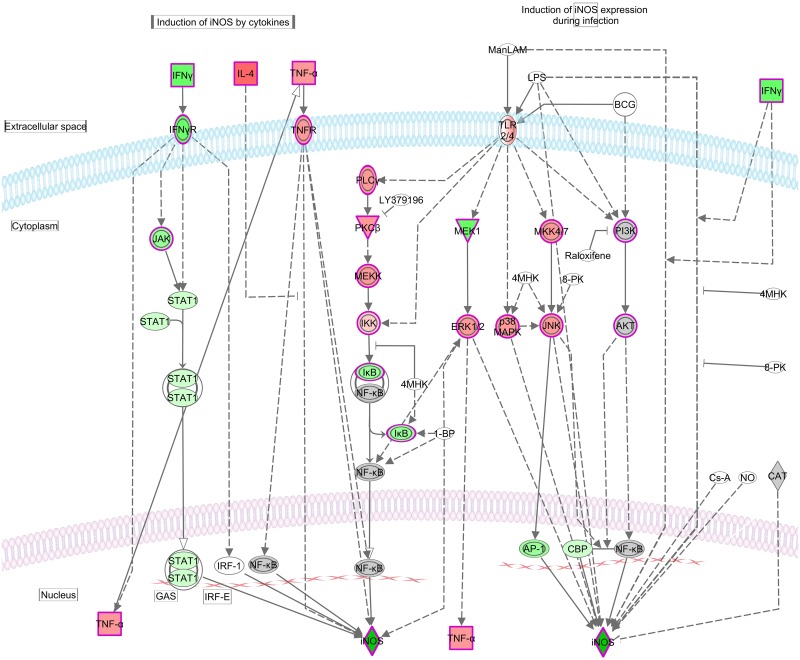
Production of nitric oxide in macrophages. This pathway is represented with gene expression (log2 fold-change) values overlaid. Red shading indicates increased expression in PBMCs at T2 compared to T1. Green shading indicates decreased expression in PBMCs at T2 compared to T1. Color intensity indicates expression level. White and grey shading indicates not significantly differentially expressed and filtered out due to low expression, respectively.

DEGS from T2vsT1 and T3vsT2 were fitted into known ‘canonical’ pathways of an IPA database to explore the potential PBMC cellular pathways modulated following *F*. *hepatica* infection. In total, 182 and 55 canonical pathways were significantly enriched at T2vsT1 and T3vsT2, respectively (FDR-adjusted *p*-value ≤ 0.05) (S6 Table). The top 10 canonical pathways from two comparisons are shown separately in Table 2. Notably, a number of these pathways in T2vsT1 are associated with innate host-defense mechanisms, including nitric oxide production in macrophages (ranked 1), IL-6 signaling (ranked 3), phagosome formation (ranked 4), and toll-like receptor signaling (ranked 10). Partial pathway graphs of ‘TGF-β signaling’ and ‘Nitric oxide production in macrophages’ are shown in Figs 4 and 5, respectively. In contrast, ‘antigen presentation’ (ranked 3) is the only adaptive immune-related pathway in the top-10 overrepresented pathways in T3vsT2. The relative expression level of selected genes from pathways of interest are presented in Table 3.

**Table 2:** Top 10 overlapped IPA canonical pathways in T2vsT1 and T3vsT2 respectively, ranked by overlap p-value.

Pathway Name	-log(p-value)*	Ratio**	z-score***
***T2vsT1***			
Production of Nitric Oxide and Reactive Oxygen Species in Macrophages	5.82	0.33	-1.291
Germ Cell-Sertoli Cell Junction Signaling	5.78	0.34	NaN
IL-6 Signaling	5.55	0.37	-1.982
phagosome formation	5.28	0.37	NaN
Role of *IL17*A in Arthritis	5.09	0.45	NaN
Xenobiotic Metabolism Signaling	5.03	0.30	NaN
Role of Macrophages, Fibroblasts and Endothelial Cells in Rheumatoid Arthritis	5.01	0.29	NaN
Death Receptor Signaling	4.93	0.38	1.521
TREM1 Signaling	4.67	0.40	-2.921
Toll-like Receptor Signaling	4.47	0.39	-1.091
***T3vsT2***			
Calcium Signaling	3.15	0.16	-0.426
Role of CHK Proteins in Cell Cycle Checkpoint Control	2.81	0.22	-0.302
Antigen Presentation Pathway	2.57	0.24	NaN
Unfolded protein response	2.37	0.20	NaN
Role of BRCA1 in DNA Damage Response	2.34	0.18	-0.632
Protein Ubiquitination Pathway	2.29	0.13	NaN
GDNF Family Ligand-Receptor Interactions	2.29	0.18	1.941
Endoplasmic Reticulum Stress Pathway	2.23	0.29	NaN
p53 Signaling	2.18	0.16	-0.775
TR/RXR Activation	2.07	0.16	NaN

*The overlap p-value indicates the probability of association of molecules from our dataset with the canonical pathway by random chance alone using Fisher’s exact test.

** In a given pathway, the ratio is calculated as number of genes in our dataset that meet the cutoff criteria, divided by the total number of genes involved in that pathway.

***The Z-score is used to indicate an overall activity status of the pathway. Z < 0 indicates a prediction of an overall decrease in activity while Z > 0 predicts an overall increase in the activity. NaN indicates pathways that are currently ineligible for a prediction.

**Table 3:** The selected gene expression level in several pathways of interest.

Pathway Name	Symbol	Description	Exp. Ratio T2/T1	adj. *p*	Exp. Ratio T3/T2	adj. *p*
*TGF-β* signaling	*TGFΒ1*	*Ovis aries* transforming growth factor, beta 1	2.231	0.023	-	-
	*COL1A1*	collagen, type I, alpha 1	14.607	0.02	10.068	0.002
	*SMAD3*	SMAD family member 3	3.775	0.005	-	-
	*SMAD4*	*Ovis aries* SMAD family member 4	4.242	1.82E-04	0.478	0.019
	*SMAD7*	SMAD family member 7	22.865	9.33E-06	-	-
	*PAI-1*	*Ovis aries* plasminogen activator inhibitor type 1	47.729	0.002	-	-
Production of nitric oxide in Macrophages	*NOS2*	nitric oxide synthase 2, inducible	5.64E-09	4.17E-06	-	-
	*IFNG*	*Ovis aries* interferon, gamma (*IFNG*), mRNA.	0.064	0.007	2.304	0.02
	*IL4*	Interleukin-4	63.181	0.001	-	-
	*STAT1*	*Ovis aries* signal transducer and activator of transcription 1	0.393	0.035	-	-
	*JAK2*	Janus kinase 2	0.217	0.002	-	-
Toll-like receptor signaling	*TLR1*	*Ovis aries* toll-like receptor 1	0.005	2.33E-06	5.057	0.001
	*TLR4*	*Ovis aries* toll-like receptor 4	3.664	0.033	0.363	0.006
	*TLR5*	*Ovis aries* toll-like receptor 5	0.036	0.014	105.282	0.014
	*TLR6*	*Ovis aries* toll-like receptor 6	0.017	9.36E-06	-	-
	*TLR7*	*Ovis aries* toll-like receptor 7	0.045	8.55E-05	3.136	0.012
	*TLR10*	*Ovis aries* toll-like receptor 10	0.002	9.93E-06	-	-
Death receptor signaling & Apoptosis signaling	*TNF*	*Ovis aries* tumor necrosis factor, *TNFα*	9.386	0.023	-	-
	*TNFRSF1A*	*Ovis aries* tumor necrosis factor receptor 1, TNFR1	4.515	0.029	-	-
	*TNFRSF1B*	tumor necrosis factor receptor 2, TNFR2	4.461	0.001	-	-
	*TNFSF12*	tumor necrosis factor (ligand) superfamily, member 12, APO3L	16.755	0.055	-	-
	*TNFRSF25*	tumor necrosis factor receptor superfamily, member 25, death receptor 3, DR3	27.218	9.94E-05	-	-
	*novel gene*	Orthologue of human tumor necrosis factor receptor superfamily, member 10a/b, death receptor 4/5, DR4/5	5.83	0.025	-	-
	*TNFRSF21*	tumor necrosis factor receptor superfamily, member 21, death receptor 6, DR6	6.005	0.021	-	-
	*FAS*	*Ovis aries* Fas (TNF receptor superfamily, member 6)	0.317	0.009	-	-
	*CYCS*	Cytochrome c	2.776	0.028	9.148	0.011
	*CASP3*	caspase 3, apoptosis-related cysteine peptidase	-	-	4.545	0.001
T helper cell differentiation & *IL17* signaling	*RORC*	RAR-related orphan receptor C	0.073	0.005	-	-
	*IL23A*	*Ovis aries* interleukin 23, alpha subunit p19	0.398	0.031	-	-
	*IL23R*	interleukin 23 receptor	3.60E-04	6.33E-05	-	-
	*IL17F*	interleukin 17F	0.017	0.002	-	-
	*IL17RC*	interleukin 17 receptor C	0.01	3.38E-04	2.074	0.038
	*novel gene*	Orthologues of human chemokine (C-X-C motif) ligand 5, CXCL5	0.005	5.02E-05	-	-
	*CCL2*	chemokine (C-C motif) ligand 2	0.012	0.001	-	-
	*CCR2*	chemokine (C-C motif) receptor 2	0.048	0.002	-	-

**Fig 6:**
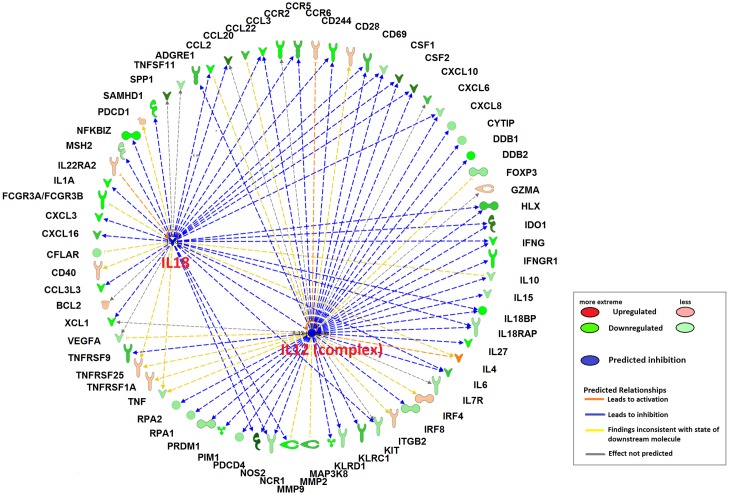
Upstream regulator analysis predicts IL18 and IL12 (complex) to be inhibited in PBMC in early infection with *F*. *hepatica*. Downstream target genes are highlighted as upregulated (red) or downregulated (green) at T2vsT1, in the symbols at the edge of the circle, with color intensity increasing with degree of fold change. The activation state of predicted upstream regulators, IL12 (complex) and IL18, is indicated as inhibited (sold blue area within the circle). Arrowheads at the end of interactions (dotted lines) indicate activation, while bars indicate inhibitory effects. The color of lines represent predicted relationships based on gene expression, including orange (activation), blue (inhibition), yellow (findings inconsistent with state of downstream molecule) and grey (effect not predicted).

DEGS and IPA upstream regulator analysis were also used to predict the upstream transcriptional regulators that may be responsible for gene expression changes observed during infection, based on prior knowledge stored in the IPA gene database. Upstream regulators are defined as any molecules that can affect the expression of another molecule, including transcription factors, cytokines and micro-RNAs. Several predicted regulators associated with the adaptive immune response are shown in Table 4. Some Th1/Th17-associated and pro-inflammatory cytokines (including IFN-γ, TNF, IL15, IL12 (complex), IL17F and IL18) as upstream regulators were predicted to be inhibited with z-score < -2. Fig 6 shows the interaction of downstream target genes regulated by IL18 and IL12 (complex), which highlight the overlapped downstream genes with the same direction of activation (e.g., IFNG, CCL3, CCR5, CD244, CSF1, CSF2, CXCL10, CXCL8, HLX, IDO1, IL18BP, IL18RAP, IL6, NCR1 and NOS2). In addition, an important regulator cytokine during *F*. *hepatica* infection, IL10, showed a trend of activation with z-score of 0.423 (overlap p-value = 3.84E-16).

**Table 4:** Predicted upstream regulators of interest at acute stage of *F*. *hepatica* infection (T2vsT1), ranked by overlap p-value.

Upstream Regulator	Exp Log Ratio	Predicted Activation State	Activation z-score*	p-value of overlap**	No. of targeted genes in dataset
*IFNG*	-3.959	Inhibited	-3.696	1.71E-19	347
*TNF*	3.23	Inhibited	-3.929	3.36E-19	413
*IL10*	-2.99		0.423	3.84E-16	125
*miR-16-5p* (and other miRNAs w/seed AGCAGCA)		Activated	2.502	9.26E-13	77
IL13			0.857	2.64E-12	122
*IL6*	-7.404		-1.427	2.28E-11	313
IL15	-3.363	Inhibited	-2.226	1.32E-10	114
*miR-155-5p* (miRNAs w/seed UAAUGCU)		Activated	2.105	5.61E-08	55
*TLR4*	1.873	Inhibited	-3.773	6.60E-08	92
*SMAD7*	4.515		0.407	2.31E-07	46
*CD40*	2.742	Inhibited	-3.047	3.42E-07	67
*CD28*	2.007	Activated	2.28	3.64E-07	96
*IL10RA*	2.592	Activated	2.881	7.15E-06	84
*MYD88*		Inhibited	-5.102	7.25E-06	70
*IL18*	-5.513	Inhibited	-3.512	1.27E-04	42
*TLR2*		Inhibited	-2.961	1.96E-04	44
*IL12* (complex)		Inhibited	-3.581	2.11E-04	56
*IL17F*	-5.848	Inhibited	-3.346	4.37E-04	13
*TLR1*	-7.669	Inhibited	-2.135	5.86E-04	10
*TLR5*	-4.803	Inhibited	-2.407	3.02E-03	14
*TLR3*		Inhibited	-3.698	3.10E-03	60
*miR-217-5p* (and other miRNAs w/seed ACUGCAU)		Inhibited	-2.412	6.32E-03	6
*TLR7*	-4.486	Inhibited	-2.697	1.59E-02	30
*TNFSF12*	4.067	Inhibited	-3.743	2.12E-02	23
*SMAD4*	2.085		1.091	3.16E-02	48

*The z-score is used to infer whether an upstream regulator is likely to be activated (z > 2) or inhibited (z < -2). It is calculated from the proportions of downstream genes which are consistent with an expected expression direction based on the known interaction between the regulator and the genes present in the IPA’s database.

**The overlap p-value indicates whether there is a statistically significant overlap between the dataset genes and the genes that are known to be regulated by a transcriptional regulator using Fisher’s exact test.

## Discussion

It is interesting that the vaccinated groups did not show any difference with the non-vaccinated groups in terms of gene expression. This would indicate that immunization did not induce any detectable alteration of the innate or adaptive immune response at transcriptomic level. The other possible explanation would be that the sample size of each group was not sufficient for detecting the vaccine effect. Therefore we focused on the biggest effect of clustering our samples—infection, by comparing the datasets from different time points. One potential missing element in this study was that we did not perform differential cell counts at each time point. However, in previous infection trials done by our group [36, 37], the hematological studies only showed an increase of eosinophils after infection of *F*. *hepatica*, but no obvious change of cell populations of PBMC during infection. Hence, we are confident that the DEG described here reflect true gene expression changes rather than cell population changes. On the other hand, even with a similar cell population before and after infection, it was still very hard to ascribe gene expression changes to a single cell type. We understand that this is a common issue for currently published RNAseq work based on bulk-sample RNAseq, such as PBMC, liver tissue, etc. However, it is reassuring that the data produced in our study is consistent with many aspects of previous observations at the protein-level in *F*. *hepatica* infection. Therefore, we believe that the data contributes to our understanding of the immunology of fasciolosis.

Compared to previous transcriptomic studies on *F*. *hepatica* infection, the work described here provides important new insights into modulation of the host immune response by *F*. *hepatica* infection. More recently, similar RNAseq work has been done on ovine PBMC following *F*. *hepatica* infection, although only extending to 8 wpi [38]. In this study, the authors identified 183 and 76 DEG at 2 and 8 wpi, respectively. Similar to our finding, they observed an up-regulated TGF-β following *F*. *hepatica* infection, and also highlighted the ‘TGF-β signaling pathway’ throughout *F*. *hepatica* infection. However, their data did not show statistically significant transcriptomic changes of a high number of genes encoding interleukins and Th2-typical cytokines (e.g. IL4) at any of either 2 or 8 wpi [38]. Here we detected 6490 (4593 DEGS with fold change ≥ 2.0) and 2364 DEGS (867 DEGS with fold change ≥ 2.0) from two comparisons respectively with FDR-adjusted p-value ≤ 0.05. These figures are much higher than the DEGS detected in previous studies [12, 13, 38]. More importantly, the findings based on our DEG datasets are also in agreement with many aspects of previous observations in fasciolosis, such as a biased Th2 responses and a suppressed Th1/Th17 responses following *F*. *hepatica* infection. We also compared DEGS expression at acute and chronic stages of ovine infection. Notably, 5398 DEGS were observed in T2vsT1 only (Fig 3), representing genes which were significantly regulated at the acute stage of infection (T2) and thereafter maintained the same expression level until the chronic stage of infection. The DEGS data provided here combined with IPA analysis allowed us to uncover potential molecular mechanisms involved in immunomodulation induced by *F*. *hepatica*. Here, several predicted upstream regulators and significantly overlapped pathways of interest are discussed.

### Potential upstream regulators affected by *F*. *hepatica* infection

It is interesting in Table 4 that the upstream regulators IL18 and IL12 were all predicted to be dramatically inhibited with z-scores of -3.52 and -3.58 respectively. Previous studies in mice have shown that the combination of IL18 and IL12 can inhibit IL4 dependent IgG1 production, and enhance IgG2a production in B cells in vivo/vitro [39]. It is known that infection of *F*. *hepatica* induce a Th2-biased immune response characterized by high titers of specific IgG1 antibodies and virtually no specific IgG2 [40, 41]. Notably, another important cytokine involved in IgG2a class switching, IL27 (down-regulated in T2*vs*T1), appears in the downstream target genes of IL12 complex (Fig 6). IL27 is produced by activated APC such as macrophages and DC [42, 43]. Previous studies have indicated that IL27 plays a role in induction of IgG2a class switching in mouse spleen B cells activated with anti-CD40 or LPS, while IL27 inhibited IgG1 class switching induced by IL4 in activated B cells [44]. Since these supporting evidences are derived from mice, future studies should be performed to investigate the role of these cytokines in IgG1/IgG2 class-switching in sheep. We hypothesize that this isotype bias seen strongly in the immune response to *F*. *hepatica* in sheep is attributable to the attenuated regulation effect of IL18 and IL12 on B cells. Three mature miRNAs were predicted to be significantly activated (miR-16-5p and miR-155-5p) or inhibited (miR-217-5p) for downstream regulation at the acute stage of infection (Table 4). Particularly, miR-155-5p is predicted to be significantly activated (z-score = 2.1; overlap p-value = 5.61E-08) with 55 predicted target genes in our dataset (such as IFNG, IL6, IL1A, TNF and chemokine ligands). The mature miR-155 (miR-155-5p) has been demonstrated to be one of the five major miRNAs that is specific for hematopoietic cells including B cells, T cells, monocytes and granulocytes [45]. MiR-155-5p has been shown to play a role in pathogen-induced immunity [46] by shaping the transcriptome of lymphoid cells that control diverse biological functions from inflammation to immunological memory [47, 48]. Therefore this lends an impetus for exploring the role of miR-155-5p in *F*. *hepatica* infection.

### Fibrosis in ovine fasciolosis

Previous studies have proposed that TGFB1 plays a central role in fibrosis during *F*. *hepatica* infection [37]. Indeed, it is well known generally that persistent TGFΒ1 signaling leads to excessive fibrosis and ultimately scarring of internal organs [49]. Through downstream SMAD signaling, TGFB1 induces the transcription of gene COL1A1, encoding collagen type I, which is the major fibrous collagen and plays a central role in wound-healing [49].

As shown in Fig 4, at the acute stage of infection, we found that TGFΒ1 and the related genes TGFBR1, SMAD 3/4 were all up-regulated, and maintained thereafter (because we didn’t detect them as DEGS in T3vsT2). Importantly the COL1A1 gene was up-regulated at the acute stage of infection (log2FC = 3.87, T2vsT1), and then rose up to an even higher level at the chronic stage (log2FC = 3.33, T3vsT2), indicating that persistent TGFΒ1 function plays a key role in accumulation of ECM (Extracellular Matrix) and fibrosis during the whole course of *F*. *hepatica* infection. This is in agreement with previous finding in the fluke-infected sheep liver tissue that genes involved in fibrosis and ECM formation (e.g. TGFΒ1 and COLIA1) were up-regulated at 8 wpi [13]. In addition, a powerful fibrosis-promoting molecule [50] plasminogen activator inhibitor-1 (PAI-1) was also up-regulated in T2vsT1 but not in T3vsT2, indicating its potential role in pro-fibrogenesis during the infection.

On the other hand, we noticed that an inhibitory-Smad protein—SMAD7—was also up-regulated in T2vsT1. SMAD7 is involved in a negative feedback loop which can suppress the fibrogenic progress mediated by SMAD2/3/4 signaling [51–53], indicating that SMAD7 may play a role in controlling fibrosis during the early stage of ovine fasciolosis. Usually during chronic liver injury, the transcription of SMAD7 is blocked by the pSmad3L pathway [54], and the lack of SMAD7 induction might lead to constitutive fibrogenic TGFΒ1 production [53]. In this study, SMAD7 expression was maintained at a similar level at the chronic stage of infection, since SMAD7 does not appear in the list of DEGS of T3vsT2. This indicates that SMAD7 may play a negative regulatory role in fibrosis formation during the chronic stage of ovine fasciolosis. Considering that fibrosis is often limited in ovine as compared with bovine fasciolosis, it would be interesting to investigate the transcription of SMAD7 during *F*. *hepatica* infection in cattle. We hypothesize that the expression level of SMAD7 in PBMC of cattle would be significantly lower at the chronic stage than that the acute stage of *F*. *hepatica* infection, while the expression of COL1A1 gene would significantly increase from acute to chronic infection.

### Down-regulation of iNOS by *F*. *hepatica* infection

NOS2, encoding inducible nitric oxide synthase (iNOS) was extremely down-regulated (log2 FC value was -27.4, p < 0.05) at the acute stage of infection, and ranked at the top of all down-regulated genes (S5 Table). This downregulation was seen also at the timepoint representing the chronic stage of infection (since NOS2 was not detected in DEGS data T3vsT2). iNOS is able to convert arginine into citrulline and nitric oxide (NO), which is considered a defense mechanism in response to pathogen invasion [55]. This result is consistent with previous observation that ovine macrophages failed to generate nitric oxide when incubated with NEJ of *F*. *hepatica* in vitro [56].

Regulation of iNOS at the transcriptional level is complex and cell- / species- specific [57]. While there are numerous transcription factors involved in iNOS expression, activation of the IFNγ-regulated transcription factor STAT-1α and thereby activation of the iNOS promoter have been demonstrated to be the essential step for iNOS induction in murine, rat and human cells, and all mammalian iNOS promoters contain several homologies with STAT-1α binding sites (GAS, Interferon-Gamma-Activated Sequence) [57, 58]. It was observed that iNOS induction in murine macrophages was blocked with a disrupted STAT1 gene [59]. In man, the inhibition of iNOS expression by various compounds has been attributed to the inhibition of the JAK-STAT-1 pathway [57]. The data described here shows that the gene expression of IFNγ, IFNγ-receptor, STAT1 and JAK were all significantly down-regulated at T2 compared to T1 (Fig 5). Although the mechanism of iNOS induction in ovine cells has not yet been described, our results indicate that the attenuated IFNγ-JAK/STAT pathway is likely account for the extreme down-regulation of iNOS during *F*. *hepatica* infection.

Previous studies have shown that *F*. *hepatica* is capable of generating alternatively-activated macrophages (AAM) characterized by having high arginase activity and low levels of iNOS [11]. We hypothesize that this dramatic down-regulation of iNOS is mainly attributed to alternative activation of macrophages during infection. Since iNOS can be expressed not only in macrophages but also in other immune cells such as CD4+ T cells [60], an interpretation of the suppression of iNOS in this study needs to consider the role of other cells in PBMC population. Previous vaccine studies have suggested that iNOS expression and subsequent NO production is important for an effective host response against the early migrating liver fluke [61]. It will be worthwhile, therefore, to investigate the relationship between the IFNγ-JAK/STAT pathway and iNOS induction in future vaccine/challenge trials with *F*. *hepatica*.

### *F*. *hepatica* comprehensively suppressed Toll-like receptor expression in PBMC

Toll-like receptors (TLR), belonging to the family of pathogen-associated pattern recognition receptors, are usually expressed in sentinel cells and play a key role in the innate immune system. Previous studies suggest that some parasites, for instance, *Leishmania*, *Entamoeba* and *Trypanosoma* can down-regulate TLR expression [62]. Our results showed that *F*. *hepatica* infection significantly downregulated the mRNA expression of TLR1, TLR5, TLR6, TLR7 and TLR10 in PBMC at the acute stage of infection (T2). The transcription of TLR1, TLR5 and TLR7 then rose again to a certain extent at the chronic stage of infection (T3) (Table 3). As the potential transcriptional regulators, the activation states of TLR1, TLR3, TLR4, TLR5 and TLR7 were predicted to be inhibited (Table 4), indicating an attenuated role of them in downstream regulation. Previous studies have suggested that helminth infection can modulate the expression level of TLR and/or their function. For example, stimulation with live microfilariae of *Brugia malayi* can significantly downregulate the mRNA expression of TLR3, TLR4, TLR5 and TLR7 in human monocyte-derived DC [63]. In cattle, the excretory/secretory products of *F*. *hepatica* restricted TLR2/TLR4-mediated activation, which may control excessive inflammatory-induced pathology during *F*. *hepatica* infection [64]. Surprisingly, in our study, TLR4 mRNA expression increased to a peak at the acute stage, then decreased to original levels at the chronic stage. In general, the present results suggest that *F*. *hepatica* may suppress TLR pathways in the host by down-regulating the expression of TLR1, 5, 6, 7 and 10, in particular during acute stage, which may be an important evasion strategy.

### Regulation of apoptosis-associated genes by *F*. *hepatica* infection

Apoptosis of immune cells has been considered as an immunosuppressive strategy used by *F*. *hepatica* during infection. [65] demonstrated in vitro that *F*. *hepatica*-derived ES was able to induce apoptosis in eosinophils (Eo) by a caspase-dependent mechanism. Further studies suggested that Fh-derived ES induce Eo apoptosis via ROS-mediated mitochondrial-membrane potential loss. In addition, *F*. *hepatica*-derived ES has also been observed to induce apoptosis in peritoneal macrophages in vitro, but the mechanism remains unclear [66].

As shown in S6 Table, the z-scores of the pathways ‘Death Receptor Signaling’ and ‘Apoptosis Signaling’ were 1.5 and 1.3 respectively in T2vsT1 (with p-value < 0.05), indicating an overall increase in the activity of both pathways in T2 compared to T1. Therefore, *F*. *hepatica* may be capable of inducing apoptosis in PBMC during infection.

In general, apoptosis is mainly induced through two distinct pathways, the death receptor pathway (extrinsic) and the mitochondrial pathway (intrinsic) [67]. For the initial step of the extrinsic apoptosis pathway, most death receptors and their corresponding ligands were up-regulated in T2vsT1 including TNF-alpha/TNFR1, TNF-alpha/TNFR2, APO3L/DR3 and death receptor 4, 5, 6, while the FAS receptor was down-regulated (Table 3; S3 Fig). TNF-α is expressed in a wide range of cells but mainly produced by macrophages [68], and is the major extrinsic mediator of apoptosis. Previous in vitro studies suggest that fluke-derived molecules such as fatty acid binding protein (Fh12) may suppress the expression of TNF-α in macrophages [69, 70]. However, this is not inconsistent with our result showing up-regulation of TNF-α expression at one wpi due to the previous observation of the mixed Th1/Th2 response at the early stage of infection. The up-regulation of both TNF-α and TNFR1/TNFR2 suggest a possibility that *F*. *hepatica* could induce the extrinsic apoptosis pathway in PBMC via TNF-TNFR, rather than by the Fas/Fasl model. In the intrinsic pathway of apoptosis, mitochondria release pro-apoptotic proteins [71]. Previous studies suggest that an activated intrinsic pathway is involved in Fh-induced Eo apoptosis, as indicated by the increase level of pro-apoptotic protein cytochrome c [10]. This is consistent with our data, in that the transcription level of the CYCS gene, encoding cytochrome c, was up-regulated at one wpi, and increased to an even higher level at 14 wpi. However, other pro-apoptotic proteins released by mitochondria were down-regulated, including DIABLO, AIFM1 and DFFB (S5 Table).

Both the extrinsic and intrinsic apoptosis pathways are caspase-dependent. Previously, it has been shown that in FhESP-induced apoptotic Eo, the activation of caspase -3, -8 and -9 is increased [10]. Caspase-8 is the major initiator in the extrinsic apoptotic pathway, and may also mediate changes in mitochondrial function [72]. Surprisingly, in our study, the transcription of Caspase-8 was down-regulated at one wpi. On the other hand, the most important executioner, caspase-3, was up-regulated at the chronic stage of infection (Table 3). There was no obvious trend in genes governing the regulation of the pro-apoptotic targets of caspases which are associated with the various morphological changes occurring during apoptosis. For example, the downstream effector genes ACTB (ACTIN) and SPTAN1 were up-regulated in T2vsT1, while LMNA, and DFFB were down-regulated, indicating a mixed-effect on apoptosis in PBMC (S5 Table; S3 Fig).

In summary, our data suggest that *F*. *hepatica* infection may have an overall role in the activation of PBMC apoptosis. Both extrinsic and intrinsic pathways may be involved and may work synergistically in infected animals. TNFα/TNFR seem to be involved in the initiation of extrinsic apoptosis. Cytochrome c may play a persistently pro-apoptotic role via the intrinsic pathway during infection. However, our data was obtained using a complex PBMC population, it is not surprising that a complex pattern of relevant gene changes was observed. Apoptosis is clearly occurring during *F*. *hepatica* infection, but it was not possible to pinpoint the exact mechanism involved. Taking into account that different mechanisms may be involved in apoptosis of diverse cell species, further study directed at the apoptosis of individual cell types is an obvious next step.

### Down-regulation of Th17 related genes by *F*. *hepatica* infection

Th17 is a relatively newly recognized subset of T helper cells distinct from Th1 and Th2 lineages, characterized by the expression of the RAR-related orphan receptor (ROR) family transcription factor RORγt, IL23 receptor (IL23R), and producing effector cytokines including IL17A, IL17F, IL21 and IL22 [73]. The role of IL17 cells in helminth-driven immune responses is still not fully understood. Studies with Schistosoma species, for example, have indicated that the Th17 response contributes significantly to severe immunopathology [74, 75], and likely in an IL23 dependent manner [76, 77]. Additionally, several studies on nematode parasites also support the robust association of Th17 and IL17 with pathology [78–80]. To date, there is no consistent evidence that Th17/IL17 have a protective effect against helminth infection [81]. In case of Teladorsagia circumcincta infection in sheep, the Th17 cytokine expression correlates with susceptibility to infection [82]. During *Schistosoma haematobium* [83] and *Echinostoma caproni* [84] infection, the Th17-related cytokines (IL17, IL21 and IL23) were associated with protection against parasite re-infection/infection respectively. Also, in a vaccination trial in mice using *F*. *hepatica*-derived synthetic peptides, the most protective peptides stimulated the production of high IFN-γ, IL4 and IL17 levels, indicating that Th17 cells may be involved in protective immune responses [85].

Here, we observed an attenuated Th17 response to *F*. *hepatica* infection with significantly down-regulated expression of the orphan retinoic acid nuclear receptor (ROR) family transcription factor RORγt, IL23/IL23R and IL17F/IL17RC (Table 3). The transcription factor RORγt plays an essential role in differentiation of Th17 cells [86]. Although IL23 is not the differentiation factor of Th17 [87], IL23 plays a fundamental role in stabilizing the Th17 lineage and expanding Th17 responses [86]. Notably, we observed a dramatic down-regulation of IL23R with a fold change of 211. Previous studies have shown that pathogenic Th17 cells exhibit a unique transcriptional signature, including high IL23R expression, distinguished from non-pathogenic Th17 cells [88]. This downregulation of IL23R in our study may indicate an attenuation of pathogenicity of Th17 cells during infection. Th17 cells are the major source of IL17/IL17A and IL17F in many types of adaptive immunity. IL17F shares the strongest sequence homology with IL17A in the IL17 family, and both cytokines promote the generation of pro-inflammatory cytokines and chemokines [89]. Importantly, dysregulated IL17A and IL17F production can result in excessive pro-inflammatory cytokine expression and chronic inflammation, leading to severe tissue damage [89]. Our data showed that one of the IL17F receptor genes, IL17RC, and its downstream chemokine gene CXCL5, were also down-regulated during *F*. *hepatica* infection (Table 3). Compared to IL17A, IL17F was found to be a more potent inducer of CXCL5 [90], a known neutrophil recruiter, indicating that *F*. *hepatica* may avoid excessive neutrophil recruitment during infection by suppressing the IL17F/CXCL5 axis.

In summary, our data suggest *F*. *hepatica* may inhibit the differentiation and stability of Th17 cells and further attenuate their role in promoting immunopathology, which in turn may benefit the parasite’s survival within the host. In addition, as Th17 responses have been proposed to be protective against *F*. *hepatica* [85], it would be interesting to investigate the correlation between the expression levels of IL17-associated genes (RORγt, IL23/IL23R and IL17F) and protection levels in future vaccination trials.

### Potential mechanisms behind immunomodulation in *F*. *hepatica* infection and its relationship with immune-mediated diseases

Epidemiological and experimental studies suggesting an inverse correlation between helminth prevalence and incidence of autoimmune diseases (summarized from [91]), indicate a protective effect of helminth infection against the development of autoimmune disease. Recent studies have demonstrated that this protective ability is associated with the suppression of host Th17 response during helminth infection [92–97]. A study in mice showed that *F*. *hepatica* can attenuate the induction of experimental autoimmune encephalomyelitis (EAE), a rodent model of human multiple sclerosis (MS), by inhibiting Th1 and Th17 responses through a TGFB-dependent mechanism [94]. The authors hypothesized that fluke-induced TGFB may suppress Th17 cells by inhibiting IL23 that would normally promote their development or expansion. Consistent with this hypothesis was the observation that two major secretory antigens of *F*. *hepatica* in their recombinant forms (rFhCL1 and rFhGST-si) suppressed the differentiation of Th17 cells by altering the function of dendritic cells (DC) to secrete reduced levels of IL-23 [98]. Our finding supports this observation, as we observed down-regulation of IL23/IL23R after infection. Previous studies have shown that IL23-deficient (IL23p19-/-) mice were resistant to EAE [99], and IL23-activated Th17 cells exhibited a higher capacity to transfer EAE than IL12-activated Th1 cells [100]. A study based on the IL-12/23 subunit (p35, p19, or p40) deficient mouse model demonstrated that the IL23/IL17 axis (Th17 response) rather than the IL12/IFNγ axis (Th1 response) is essential for the establishment of EAE [99]. The importance of the IL-23/IL17 axis is also supported in human MS [101]. In addition, a chemokine-chemokine receptor system CCL2 (or MCP-1)-CCR2 pathway has been proposed to be essential for development of EAE/MS as this signaling pathway might play an important role in migration of Th17 cells to MS lesions [101]. This is supported by the recent observation that Th17-expressed CCR2 drives Th17 recruitment to the inflamed central nervous system (CNS) during EAE [102]. Interestingly, our data showed a significant down-regulation of CCR2 and its ligand CCL2 induced by *F*. *hepatica* infection (Table 3), suggesting a potential role of *F*. *hepatica* in inhibition of Th17 recruitment into EAE/MS lesions. We hypothesize that *F*. *hepatica* infection may attenuate EAE/MS by suppressing these key genes involved in the IL23/IL17 axis and CCL2/CCR2 signaling pathways. Further identification of specific, or combinations of *F*. *hepatica*-derived molecules involved in suppressing these genes would be for important for further investigation of their immunotherapeutic potential against human MS and other autoimmune diseases.

In conclusion, this is one of the first study which describes gene expression changes of host PBMC in response to *F*. *hepatica* infection. The study was carried out in sheep, which, along with cattle, represent one of the two major livestock species that are the targets for development of vaccines as a control measure for fasciolosis. Overall, our study revealed a Th2-biased immune response to infection, and suppression of Th1/Th17 responses, as expected. The potential regulatory ability of IL12, IL18 and miR155-5p in PBMCs during *F*. *hepatica* infection were proposed and further investigation is warranted. Notably, based on the analysis of the data, we hypothesize that up-regulated TGFβ1, involved with induction of fibrosis during infection, may work through SMAD2/3/4 signaling. The inhibitory-Smad protein (SMAD7) may play a key role in limitation of fibrosis formation in ovine fasciolosis. The comprehensive down-regulation of Toll-like receptors (e.g. TLR1, 5, 6, 7 and 10) and up-regulation of death receptors (e.g. TNFR1, TNFR2, DR3, 4, 5 and 6) was observed in PBMC obtained from sheep during the early stage of infection, and this indicates that *F*. *hepatica* may attenuate the inflammatory response through altering the function of sentinel cells and inducing apoptosis in PBMC. Finally, *F*. *hepatica* likely inhibits the differentiation and stability of Th17 cells through down-regulation of the transcriptional factor RORγt and IL23/IL23R. The dataset provided here, from one of the major target livestock species provides information pertinent to understanding of the immune response to fasciolosis, as well as a base from which to further refine development of vaccines. This study also shed a light on helminth-mediated immunoregulation as it may impact on control of allergic and immune-mediated diseases of man and animals.

## Supporting Information

S1 Table Parameters used in STAR. (XLSX)

S2 Table Primer-design and qPCR results. (XLSX)

S3 Table Summary information of alignment of 32 libraries. (XLSX)

S4 Table Thirty-six DEGS from vaccinated versus control animals. (XLSX)

S5 Table DEGS list of T2vsT1 and T3vsT2, respectively. (XLSX)

S6 Table Significantly overlapped IPA canonical pathways in T2vsT1 and T3vsT2, respectively. (XLSX)

S1 Fig **(A) Number of *Fasciola hepatica* recovered from the liver at necropsy. (B) Faecal egg counts at 10 and 16 wpi.** For both graphs, dots represent individual animals. Lines indicate means per group +/- SEM. ‘Vacc’ means vaccinated group. ‘Ctrl’ means control group. Differences between groups were tested using the Mann–Whitney U test. A p value of <0.05 was considered statistically significant. The results showed no significant difference between vaccinated and control groups in terms of neither fluke burden nor faecal egg counts.
(TIF)

S2 Fig QC plot showing the expression density plot for the individual samples in each time point after filtering. Based on this plot there is no evidence of any poor quality or outlier libraries and aggressive normalization (e.g. quantile normalization) was not required.
(TIF)

S3 Fig Death receptor signaling pathway. This pathway is represented with gene expression (log_2_ fold-change) values overlaid. Red shading indicates increased expression in PBMCs at T2 compared to T1. Green shading indicates decreased expression in PBMCs at T2 compared to T1. Color intensity indicates degree of expression level. White and grey shading indicates not significantly differentially expressed and filtered out due to low expression respectively.
(TIF)
