# Supplementary figures and images for: Transcriptomic Study on Ovine Immune Responses to Fasciola hepatica Infection
Source: PLoS Negl Trop Dis. 2016 Sep 23;10(9):e0005015. doi: 10.1371/journal.pntd.0005015 (PMC5035020; doi:10.1371/journal.pntd.0005015)

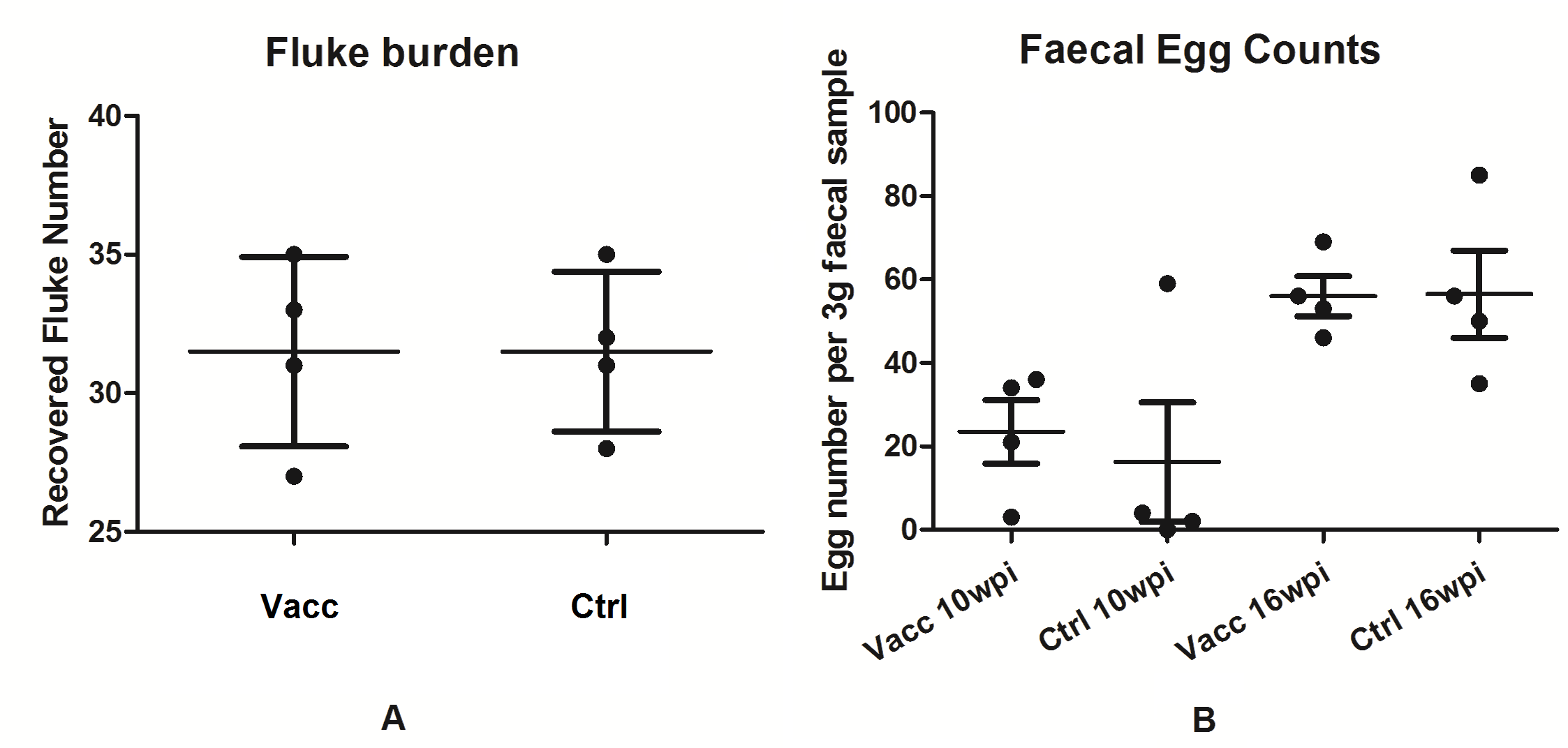

Supplement: S1 Fig — (A) Number of Fasciola hepatica recovered from the liver at necropsy. (B) Faecal egg counts at 10 and 16 wpi. For both graphs, dots represent individual animals. Lines indicate means per group +/- SEM. ‘Vacc’ means vaccinated group. ‘Ctrl’ means control group. Differences between groups were tested using the Mann–Whitney U test. A p value of <0.05 was considered statistically significant. The results showed no significant difference between vaccinated and control groups in terms of neither fluke burden nor faecal egg counts. (TIF) [file pntd.0005015.s007.tif]

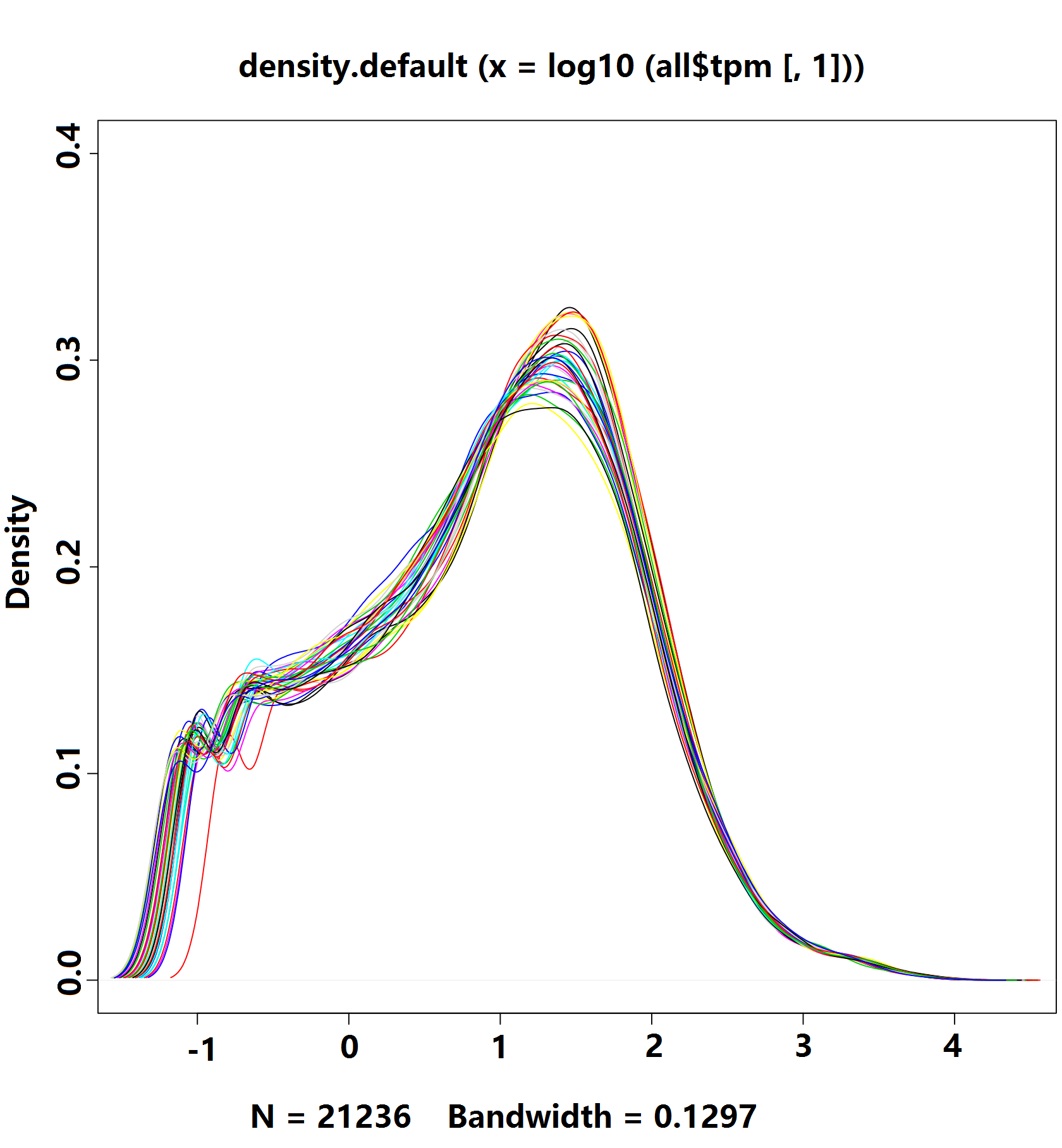

Supplement: S2 Fig — Based on this plot there is no evidence of any poor quality or outlier libraries and aggressive normalization (e.g. quantile normalization) was not required. (TIF) [file pntd.0005015.s008.tif]

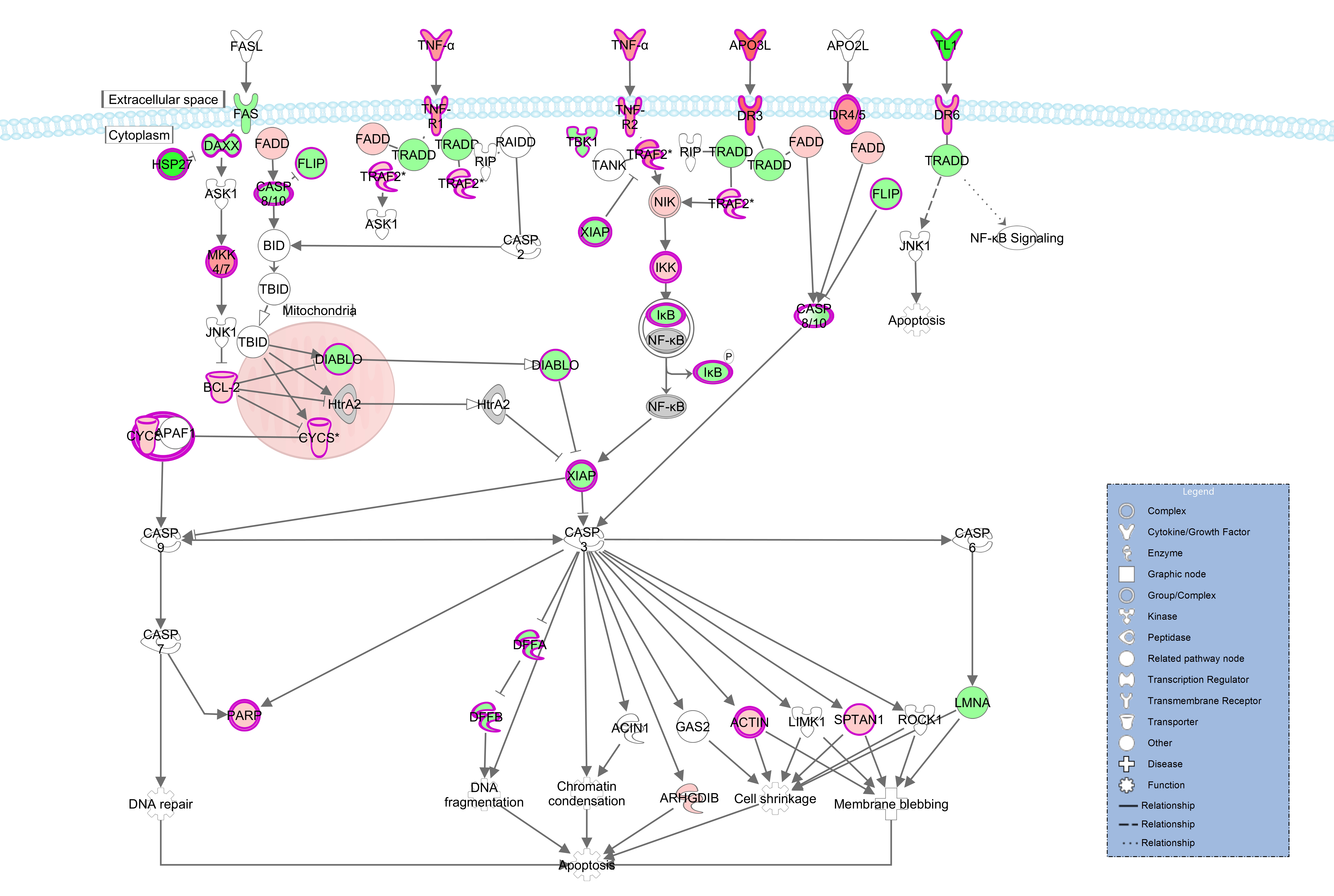

Supplement: S3 Fig — This pathway is represented with gene expression (log2 fold-change) values overlaid. Red shading indicates increased expression in PBMCs at T2 compared to T1. Green shading indicates decreased expression in PBMCs at T2 compared to T1. Color intensity indicates degree of expression level. White and grey shading indicates not significantly differentially expressed and filtered out due to low expression respectively. (TIF) [file pntd.0005015.s009.tif]
